# Elderly Use Proprioception Rather than Visual and Vestibular Cues for Postural Motor Control

**DOI:** 10.3389/fnagi.2015.00097

**Published:** 2015-06-23

**Authors:** Isabella Katharina Wiesmeier, Daniela Dalin, Christoph Maurer

**Affiliations:** ^1^Klinik für Neurologie und Neurophysiologie, Universität Freiburg, Freiburg, Germany

**Keywords:** elderly, postural control, proprioception, vestibular, model

## Abstract

Multiple factors have been proposed to contribute to the deficits of postural control in the elderly. They were summarized as sensory, motor, and higher-level adaptation deficits. Using a model-based approach, we aimed to identify which of these deficits mainly determine age-related changes in postural control. We analyzed postural control of 20 healthy elderly people with a mean age of 74 years. The findings were compared to data from 19 healthy young volunteers (mean age 28 years) and 16 healthy middle-aged volunteers (mean age 48 years). Postural control was characterized by spontaneous sway measures and measures of perturbed stance. Perturbations were induced by pseudorandom anterior–posterior tilts of the body support surface. We found that spontaneous sway amplitude and velocity were significantly larger, and sway frequencies were higher in elderly compared to young people. Body excursions as a function of tilt stimuli were clearly different in elderly compared to young people. Based on simple feedback model simulations, we found that elderly favor proprioceptive over visual and vestibular cues, other than younger subjects do. Moreover, we identified an increase in overall time delay challenging the feedback systems stability, and a decline in the amplitude of the motor feedback, probably representing weakness of the motor system. In general, these parameter differences between young and old may result from both deficits and compensation strategies in the elderly. Our model-based findings correlate well with deficits measured with clinical balance scores, which are widely used in clinical practice.

## Introduction

Postural control in elderly people is impaired by numerous factors [for an overview, see, e.g., Shumway-Cook and Woollacott (2001) and Iosa et al. (2014)]. Changes in sensory systems include a reduced joint position sense at the ankle (Horak et al., 1989; Goble et al., 2009), paralleled by a higher perception threshold for vibration (Tang and Woollacott, 1996; Hilz et al., 1998; Lin et al., 2005; Shaffer and Harrison, 2007). Moreover, visual function (visual acuity, contour and depth perception, contrast sensitivity, peripheral vision) is reduced, partly due to structural changes of the eye. In addition, a decrease of vestibular function has been described (Bergström, 1973; Rosenhall, 1973; Merchant et al., 2000; Park et al., 2001; Rauch et al., 2001; Shumway-Cook and Woollacott, 2001; Nag and Wadhwa, 2012; Grossniklaus et al., 2013).

Impairments of the motor system in the elderly have numerously been reported [e.g., Doherty (2003), Macaluso and De Vito (2004), and Reeves et al. (2006)]. For example, a 40% reduction of the lower body muscle strength was found when compared to young healthy adults (Shumway-Cook and Woollacott, 2001). During balance corrections, elderly people display an altered muscle response organization (Shumway-Cook and Woollacott, 2001; Tsai et al., 2014) and more frequent coactivations of antagonist muscles (Shumway-Cook and Woollacott, 2001; Macaluso and De Vito, 2004; Klass et al., 2007; Papegaaij et al., 2014).

Some authors proposed deficits in higher-level adaptive systems [e.g., Shumway-Cook and Woollacott (2001)]. They suggested that elderly people’s ability to adapt to external perturbations is diminished (Horak et al., 1989; Peterka and Black, 1990; Mansfield and Maki, 2009). Elderly people react with longer onset latencies to external perturbations than young adults do (Woollacott et al., 1988; Horak et al., 1989; Woollacott and Shumway-Cook, 1990; Tsai et al., 2014). In addition, elderly people seem to have difficulties in sensory reweighting (Horak et al., 1989; Teasdale and Simoneau, 2001; Eikema et al., 2012, 2014). The term “sensory reweighting” was established by Nashner et al. (1982) to describe a process of scaling the relative importance of sensory cues (visual, vestibular, and proprioceptive) for motor control (Nashner et al., 1982; Jeka et al., 2006). However, the sensory weighting process itself seems to be unimpaired in elderly people (Allison et al., 2006; Jeka et al., 2006), indicating that differences in sensory weights between elderly and young people are related to different sensory preferences.

Measures of human postural control are usually segregated into spontaneous sway measures and measures of motor behavior induced by external perturbations. Age-related differences in spontaneous sway mainly concern increases in mean velocity (MV) and mean frequency (MF) (Maki et al., 1990; Hytönen et al., 1993; Baloh et al., 1994; Collins et al., 1995; Prieto et al., 1996; Maurer and Peterka, 2005; Qu et al., 2009).

During perturbed stance, somatosensory cues affect postural control in young people differently than in elderly people [e.g., Peterka and Black (1990), Speers et al. (2002), Fransson et al. (2004), Ghulyan et al. (2005), and Maitre et al. (2013)]. In general, stance of elderly people is reported to be less stable with absent or altered proprioceptive, vestibular, and visual information (Peterka and Black, 1990; Whipple et al., 1993; Speers et al., 2002; Rosengren et al., 2007; Liaw et al., 2009; Pierchała et al., 2012; Maitre et al., 2013; Eikema et al., 2014), leading to larger body excursions. Some authors evaluated the relationship between the perturbation and the induced body motion in terms of transfer functions [see Materials and Methods, see also Nashner and McCollum (1985), Ishida et al. (1997), Van der Kooij et al. (2001), Peterka (2002), Maurer and Peterka (2005), Masani et al. (2006), Maurer et al. (2006a), Vette et al. (2010), Davidson et al. (2011), Nishihori et al. (2012), and Van der Kooij and Peterka (2011)]. Transfer functions are frequently interpreted using a model-based approach. These models usually involve inverted pendulum bodies, a neural controller including a proportional (stiffness of the system) and a derivative feedback gain (damping of the system), and a feedback time delay. The proportional gain is proportional to the sensory error signal and the derivative gain is proportional to the time derivative of the sensory error signal. Both factors are added up as a motor output to provide corrective ankle torque, thereby stabilizing the inverted pendulum and reducing oscillations (Peterka, 2002; Vette et al., 2010). The feedback time delay represents the lumped time delays of sensory, central, and motor transduction (Peterka, 2002). The sensory weighting mechanism of the model scales the gains of the sensory cues (proprioceptive, vestibular, and visual) in terms of relative contributions to the overall feedback gain (Maurer et al., 2006a; Van der Kooij and Peterka, 2011; Engelhart et al., 2014).

Recently, some authors applied the model-based approach to postural control data of elderly people (Cenciarini et al., 2009, 2010; Davidson et al., 2011; Nishihori et al., 2012; Maurer and Peterka, 2005). During quiet stance, elderly people seem to have an increased proportional gain of the sensorimotor control system (Maurer and Peterka, 2005; Nishihori et al., 2012). During external perturbations, an increased derivative gain of the system has been reported (Cenciarini et al., 2009, 2010; Davidson et al., 2011), whereas reports of the system’s proportional gain were controversial (Cenciarini et al., 2009, 2010; Davidson et al., 2011), depending, for example, on the direction of sway.

In the current study, we aimed to find out whether we are able to detect sensory, motor, and higher-level adaptation deficits mentioned above in postural control behavior of the elderly. In addition, we aimed to identify which of these contributors most significantly influence postural control in the elderly. For that, we assessed both, spontaneous sway parameters and applied external perturbations in young, middle-aged, and elderly people. The subjects’ reactions to anterior–posterior platform tilts were analyzed at 11 frequencies with eyes closed or open, using different amplitudes, with the purpose to simultaneously identify the major components of the sensorimotor control system and their modifications as a function of age. We hypothesized that degradations of sensory, motor, and higher-level adaptation deficits in elderly could be extracted from postural control behavior. As our model-based approach is highly sensitive to changes in the sensory-motor system, it might be valuable in future for differentiating between age-related and pathology-related impairments of postural control, and for evaluating therapeutic interventions that ameliorate postural control in elderly.

## Materials and Methods

Subjects were tested by recording spontaneous sway as well as motor reactions to platform tilts (perturbed stance). In addition, elderly subjects were tested using relevant standardized clinical tests.

### Subjects

In this study, we measured postural control of elderly people (60–80 years group) and compared their data with data from middle-aged (40–59 years group) and young (20–39 years group) people.

The group of elderly subjects consisted of 20 participants with a mean age of 74 ± 3.4 years (Mean ± SD, 10 female, 10 male). We excluded elderly people suffering from any disease that may interact with postural control. For that, each subject was carefully examined by a senior consultant neurologist. In addition, we specifically tested for vestibular function using Frenzel goggles on a turning chair. We thereby quantified the function of the vestibular–ocular reflex. Moreover, we evaluated proprioceptive function, which is related to deep sensibility, by testing for position sense and by quantifying vibration sense using a vibrating tuning fork. Further exclusion criteria included any acute or chronic disease that may influence the general condition of health.

Elderly subjects were also assessed using the timed up and go test (TUG, Enkelaar et al., 2013) and the functional reach test (FRT, Enkelaar et al., 2013). Our emphasis was to monitor elderly people’s balance function with widely accepted clinical tests. Furthermore, we correlated those relevant clinical tests with our postural control measurements to evaluate whether functional impairments shown by one of our parameters may be linked to abnormalities in clinical tests.

For comparison, we analyzed data of 16 healthy volunteers (middle-aged group) between 36 and 58 years with a mean age of 48.2 ± 5.3 years (9 female, 7 male) who had been measured in our laboratory during the previous years. In addition, data of 19 healthy volunteers (20–39 years group) between 22 and 34 years with a mean age of 27.6 ± 3.7 years (10 female, 9 male) were acquired.

### Procedures

**Figure 1:**
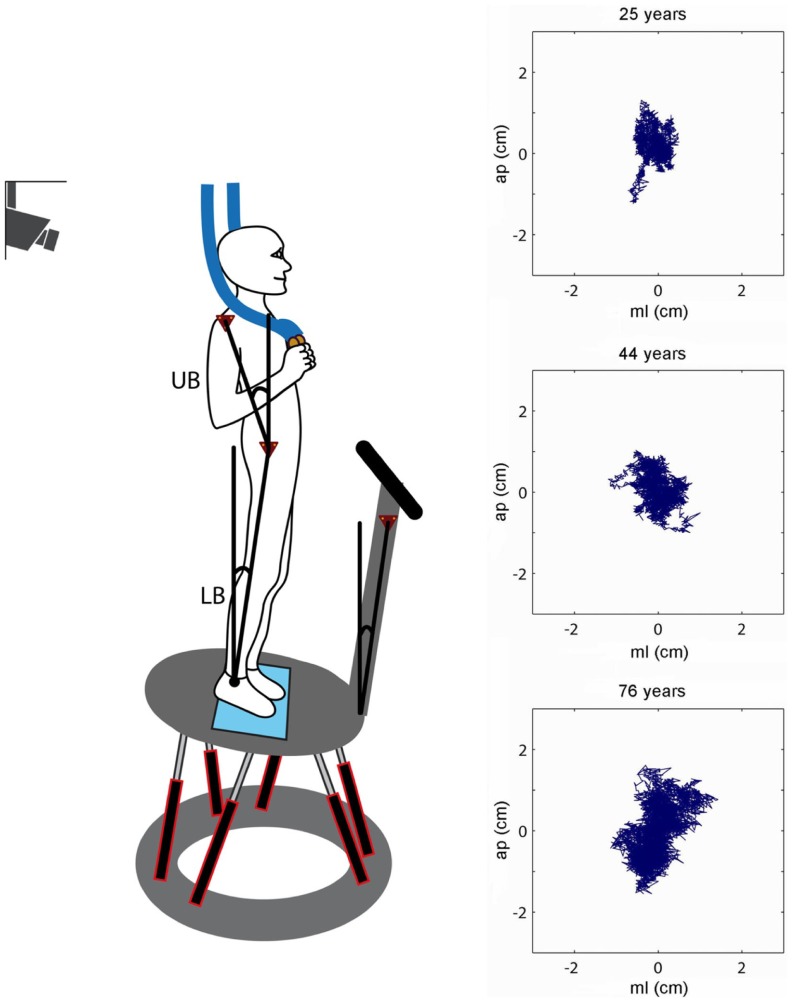
**Experimental setup**. Scheme of a participant who stands on the platform in an upright position. The six linear motors rotated the platform in the sagittal plane about the axis of the ankle joints. Angular excursions of the upper body (UB) and lower body (LB) and the platform in space were measured using an optoelectronic motion-measuring device with markers attached to shoulder, hip, and platform. Sway paths of a young (25 years), middle-aged (44 years), and elderly person (76 years) are shown on the right (ap, anterior–posterior; ml, medio-lateral).

Spontaneous sway and perturbed stance were measured on a custom-built motion platform (Cnyrim et al., 2009; Figure 1). For that, participants were told to stand upright in a relaxed position on the platform, wearing their normal shoes. Stance width was predetermined within a marked area (maximum 30 cm). For safety reasons, participants held two ropes hanging from the ceiling in a way they were not able to attain any orientation cues (Cnyrim et al., 2009). However, no subject fell, probably due to the small stimulus amplitudes. Spontaneous sway was measured on the non-moving platform with eyes open and with eyes closed. Each trial lasted 1 min. Between each trial, a short break was taken according to the participants needs.

We measured center-of-pressure (COP) sway paths and 3-D angular positions of the body in order to calculate spontaneous sway measures (parameters) and measures of perturbed stance. The COP sway path was detected with the help of a force transducing platform (Kistler platform type 9286, Winterthur, Switzerland, Figure 1). 3-D angular positions (Angular excursions) of the body (hip-to-ankle, shoulder-to-hip) and the platform in space were measured using an optoelectronic motion-measuring device with markers attached to shoulder, hip, and a rigid bar solely fixed to the platform (Optotrak 3020, Waterloo, ON, Canada). Each marker consisted of three light-emitting diodes fixed to a rigid triangle. 3-D angular positions of the triangles were used to calculate marker positions (Maurer et al., 2006b). Optotrak^®^ and Kistler^®^ output signals as well as the stimulus signals were transferred on-line to a computer system (IBM compatible Pentium^®^) via an analog-digital converter at a sampling rate of 100 Hz. We recorded all data with software programed in LabView^®^ (National Instruments, Austin, TX, USA). Center of mass (COM) height above the ankle joints was calculated according to tables from Winter (1995) using the measured heights of hip and shoulder markers. A detailed description of the experimental setup has been published previously (Maurer et al. 2003, 2006a; 2006b; Cnyrim et al., 2009).

**Figure 2:**
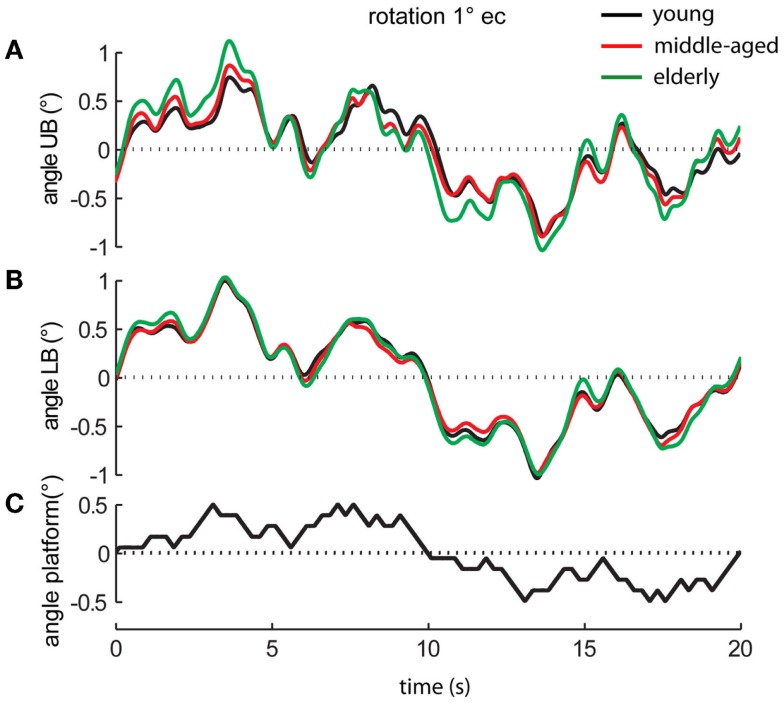
**Platform movements and motor reactions of the human body over time**. Motor reactions of the three age groups’ **(A)** upper body (UB) and **(B)** lower body (LB) at 1° stimulus amplitude with eyes closed (ec) and **(C)** platform movement (black bottom line).

Perturbed stance was measured on the moving platform with eyes open and with eyes closed (Figure 2). The rotational tilt is characterized by a platform rotation in the sagittal plane with the tilt axis passing through the participant’s ankle joints (Maurer et al., 2006a,b; Cnyrim et al., 2009). Platform rotations were designed as pseudorandom stimuli (PRTS, pseudorandom ternary sequence) with 2 peak angular displacements (0.5° and 1°) at 11 frequencies (0.05, 0.15, 0.3, 0.4, 0.55, 0.7, 0.9, 1.1, 1.35, 1.75, and 2.2 Hz).

### Data analysis

Data analysis was performed off-line with custom-made software programed in MATLAB^®^ (The MathWorks Inc., Natick, MA, USA; Maurer et al., 2003, 2006b; Cnyrim et al., 2009). From the lower and upper body excursions and COP over time in anterior–posterior (and medio-lateral directions), we calculated root mean square (RMS) around the mean COP position (Prieto et al., 1996; Maurer et al., 2003).

$$\text{RMS}=\text{SD}=\sqrt{\frac{1}{n}\sum_{i=1}^{n}{\left(x_{\text{COP}}\left(i\right)\right)}^{2}}$$

Mean velocity is the average of the absolute COP velocity (Maurer and Peterka, 2005). It was calculated by differentiating the corresponding time series.

$$\text{MV}=\frac{1}{n-1}\sum_{\text{i}=1}^{\text{n-1}}\left|{\dot{x}}_{\text{COP}}\left(i\right)\right|$$

Mean frequency was computed as the ratio of MV and mean distance (MD).

$$\text{MFREQ}=\frac{\text{MV}}{4\cdot \sqrt{2}\cdot \text{MD}}$$

Further details on spontaneous sway measures can be found in Maurer and Peterka (2005).

**Figure 3:**
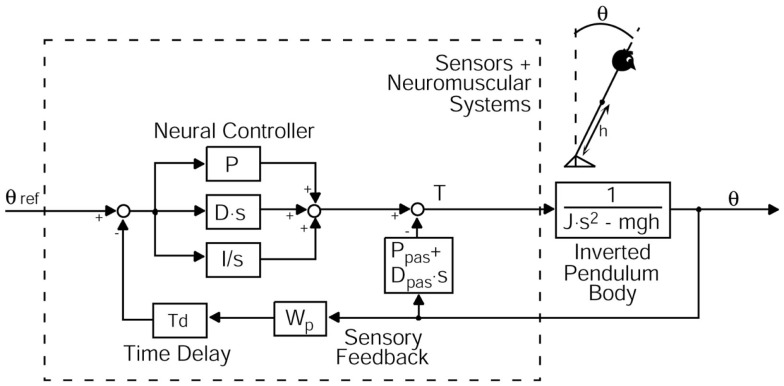
**Modified postural control model describing perturbed stance**. The model consists of a body represented by an inverted pendulum with the mass concentrated at the center of mass (COM) of the body and the sensors and neuromuscular systems including a Neural Controller. θ, body sway angle; h, height of the COM above the ankle joints; θ ref, external stimulus; P, proportional gain (stiffness factor), D, derivative gain (damping factor), I, integral gain of the neural controller; Ppas, passive stiffness factor; Dpas, passive damping factor; Wp, proprioceptive sensory weight; Td, feedback time delay; T, control torque; J, moment of inertia of the body; mgh, body mass × gravitational constant × height of the COM from the ankle joint; s, Laplace transform variable.

Transfer functions from stimulus–response data were calculated by a discrete Fourier transform (Peterka, 2002; Cnyrim et al., 2009). Fourier coefficients of stimulus and response time series are used to determine GAIN and PHASE with respect to stimulus frequencies (Maurer et al., 2006a). GAIN (response sensitivity) shows the relationship between the platform angle (stimulus amplitude) and the lower body or upper body response amplitude (Peterka, 2002; Maurer et al., 2006a; Cnyrim et al., 2009). PHASE is the relative delay between the stimulus and the reaction of the body. The transfer functions were used as the experimental data basis for model simulations using a predefined model of upright stance (see Figure 3).

Statistics were performed using Microsoft Excel and statistic programs (JMP^®^ and Statview by SAS Institute Inc., Cary, NC, USA). Statistical significance was tested by a two-level analysis of variance (ANOVA) unless stated otherwise. The between-subjects factor was age, the within-subjects factors were visual condition, sway direction, stimulus amplitude, stimulus frequency, and body segment (hip, shoulder). The level of statistical significance was set at *p* = 0.05. For the elderly group, relationships between clinical test parameters and parameters obtained from platform experiments were analyzed using a Pearson Correlation [see Maurer and Peterka (2005)]. We created a matrix of correlation coefficients, which depicts the strength of linear relationships between each pair of parameters.

The study was performed according to the ethical standards of the Declaration of Helsinki. It was approved by the ethics committee of the University of Freiburg. All participants gave their written informed consent prior to study participation.

### Model simulations

We used a specific modification of an established postural control model (Van der Kooij et al., 2001; Mergner et al., 2002, 2003; Peterka, 2002; Maurer et al., 2004, 2006a; Cnyrim et al., 2009; Engelhart et al., 2014) to extract relevant parameters of postural control. This model includes a negative feedback loop that relates body excursion detected by visual, vestibular, and proprioceptive sensors to a corrective torque via a neural controller with proportional [P], derivative [D], and integral [I] contributions (PDI-controller, Figure 3). Neural controller gains are, in part, determined by mass and height of the COM of the individual subject [see Peterka (2002) and Cenciarini et al. (2010)]. Because our elderly group displayed lower masses and heights, we had to correct neural controller gains for this effect. That is why we give numbers for [P/mgh], [D/mgh], and [I/mgh], where (mgh) represents the gravitational pull (body mass) × (gravitational constant) × (height of COM from the ankle joint). [P/mgh] and [D/mgh] represent the stiffness and damping of our model. The proportional gain [P/mgh], the derivative gain [D/mgh], and the integral gain [I/mgh] simulate the regulatory activity of the central nervous system to perturbations of stance (Nishihori et al., 2012).

Moreover, the model contains a lumped time delay [Td], which represents the time interval between the stimulus and the motor reaction. In addition, the model includes a sensory weighting mechanism [Wp], which represents the coordinate frame of the body excursion (visual and vestibular coordinates vs. platform coordinates). [Wp] describes the contribution of proprioception to the sensory feedback (Peterka, 2002). A decrease in [Wp] signifies that people tend to rely less on proprioception and more on vestibular and/or visual feedback. The biomechanics part represents the passive elasticity [Ppas] and passive damping factor [Dpas] of the muscles and tendons (Figure 3). With the help of an optimization procedure (fmincon/Matlab, Mathworks), we fit the model-derived transfer functions to the experimental transfer functions (GAIN and PHASE values) under different stimulus amplitudes and visual conditions. Subsequently, the set of model parameters representing the optimal fit were read out.

## Results

### Spontaneous sway

**Figure 4:**
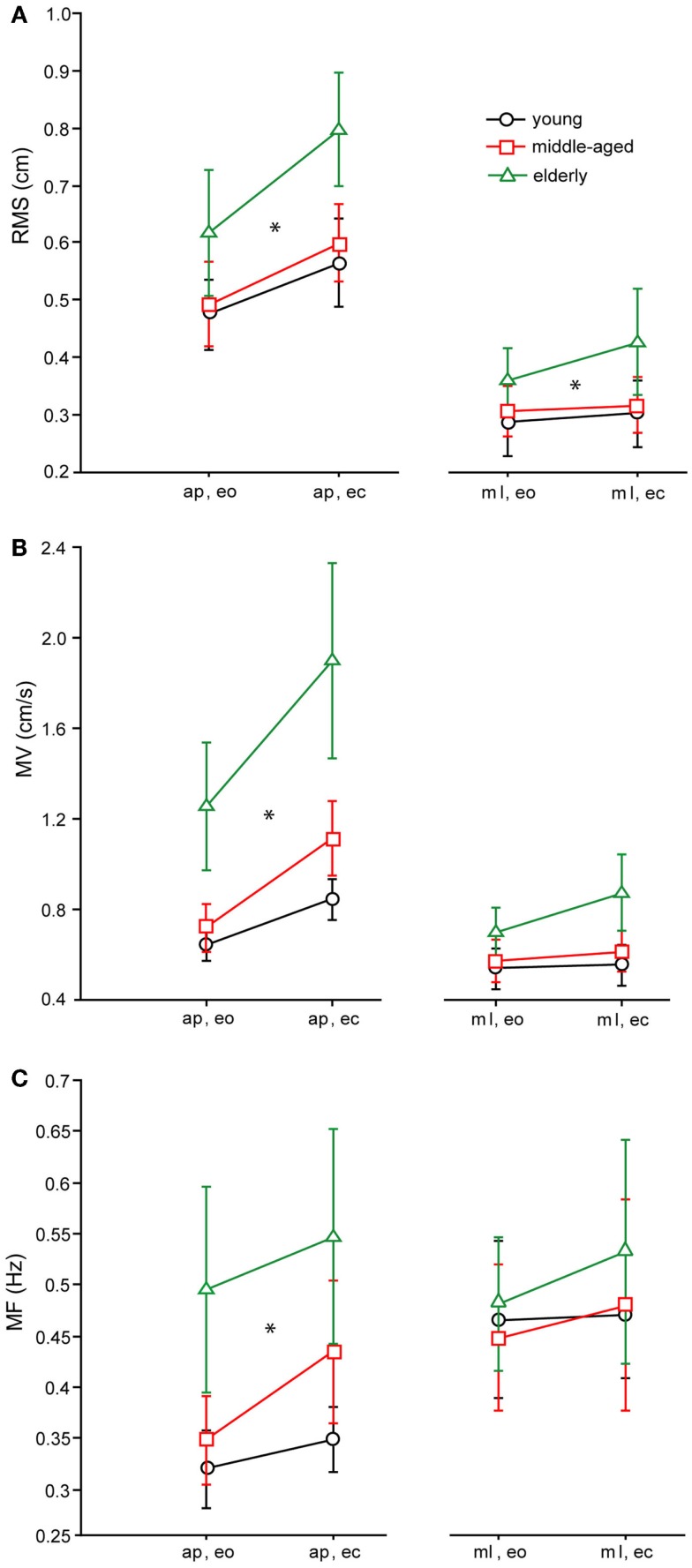
**Spontaneous sway parameters derived from COP traces**. **(A)** Root mean square (RMS) of the three age groups, **(B)** mean velocity (MV) of the THREE age groups, **(C)** mean frequency (MF) of the three age groups. a–p, anterior–posterior; ml, medio-lateral; eo, eyes open; ec, eyes closed.

The RMS of the elderly group [0.56 ± 0.011 cm, (Mean ± Standard Error)] was significantly larger than the RMS of the middle-aged (0.452 ± 0.011 cm) and young group (0.447 ± 0.011 cm, *F* = 22.98, *p* < 0.0001, see Figure 4A for the COP-derived measures). Across all age groups, RMS was larger in the anterior–posterior (a–p) direction (0.60 ± 0.01 cm) than in the medio-lateral (m–l) direction (0.38 ± 0.01 cm; *F* = 187.5, *p* < 0.0001), and significantly larger with eyes closed (ec, 0.52 ± 0.01 cm) than with eyes open (eo, 0.45 ± 0.01 cm; *F* = 19.3, *p* < 0.0001). In general, RMS of the shoulder (0.55 ± 0.011 cm) was significantly larger than RMS of the hip (0.42 ± 0.011 cm, *F* = 66.12, *p* < 0.0001). There were no significant interactions between age group and sway direction (*F* = 0.97, *p* = 0.38), visual condition (*F* = 1.38, *p* = 0.25), and body segments (*F* = 1.52, *p* = 0.22).

As with RMS, MV of the elderly group was significantly higher (0.7 ± 0.018 cm/s) than MV of the middle-aged (0.46 ± 0.02 cm/s) and young group (0.41 ± 0.018 cm/s, *F* = 66.8, *p* < 0.0001, see Figure 4B for the COP-derived measures). Across all age groups, MV was larger in the a–p direction (0.65 ± 0.015 cm/s) than in the m-l direction (0.4 cm/s; *F* = 122.7, *p* < 0.0001), and significantly larger with eyes closed (0.6 ± 0.015 cm/s) than with eyes open (0.45 ± 0.015 cm/s; *F* = 38.7, *p* < 0.0001). In addition, across all age groups, MV of the shoulder was significantly larger (0.4 ± 0.019 cm/s) than MV of the hip (0.3 ± 0.019 cm/s, *F* = 47.5, *p* < 0.0001). Age group and sway direction significantly interacted, representing the fact that the MV difference between age groups was much larger in the a–p than in the m-l direction (*F* = 4.2, *p* = 0.0153). Furthermore, there were no significant interactions between age group and visual condition (*F* = 1.93, *p* = 0.146), or between age group and body segments (*F* = 1.3, *p* = 0.285).

Effects on MF were similar to the effects on MV (see Figure 4C for the COP-derived measures). The MF of the elderly group was significantly higher (0.3 ± 0.007 Hz) than the MF of the middle-aged (0.25 ± 0.007 Hz) and the young group (0.24 ± 0.007 Hz, *F* = 16.97, *p* < 0.0001). Across all age groups, MF was larger in the m-l direction (0.28 ± 0.006 Hz) than in the a–p direction (0.25 ± 0.006 Hz, *F* = 3.4, *p* = 0.035), and significantly larger with eyes closed (0.28 ± 0.006 Hz) than with eyes open (0.25 Hz; *F* = 9.18, *p* = 0.003). In general, MF of hip (0.17 ± 0.007 Hz) and shoulder (0.17 ± 0.007 Hz) were nearly equal, which was not statistically significant (*F* = 0.2, *p* = 0.620). Age group and sway direction significantly interacted (*F* = 3.4, *p* = 0.035), representing the fact that the MF difference between age groups was much larger in the a–p than in the m-l direction. In addition, we found no significant interaction between age group and body segments (*F* = 0.1, *p* = 0.887) or between age group and visual condition (*F* = 1.9, *p* = 0.157).

### Externally perturbed stance

We characterize the participants’ sway behavior as a function of external perturbation by a transfer function in the frequency domain over 11 frequencies (0.05–2.2 Hz, see Procedures). The transfer function consists of a gain and a phase curve. For didactic reasons, we display GAIN effects of the middle-aged and elderly group also as GAINFACTOR with respect to a reference group (young group). GAINFACTOR is the percentage gain of the two elder groups with respect to the young reference group.

**Figure 5:**
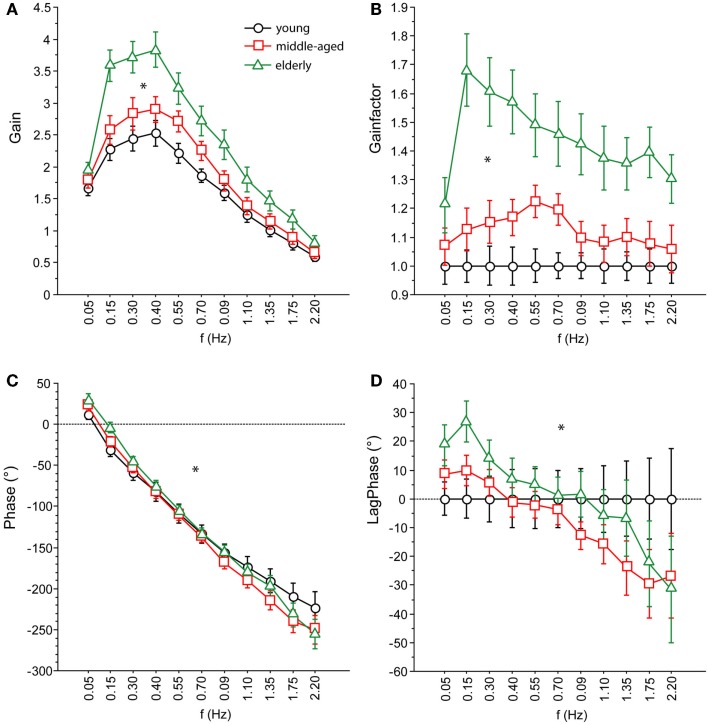
**Parameters of perturbed stance**. **(A)** GAIN, **(B)** GAINFACTOR, **(C)** PHASE, **(D)** LAGPHASE of the three age groups across all stimulus amplitudes and visual conditions. GAINFACTOR is the percental quotient between the GAIN of the middle-aged and elderly group and the young group as a comparison group. The time difference between PHASE of the middle-aged and elderly group and the young group as a comparison group is named LAGPHASE. Its unit is degrees (°).

**Figure 6:**
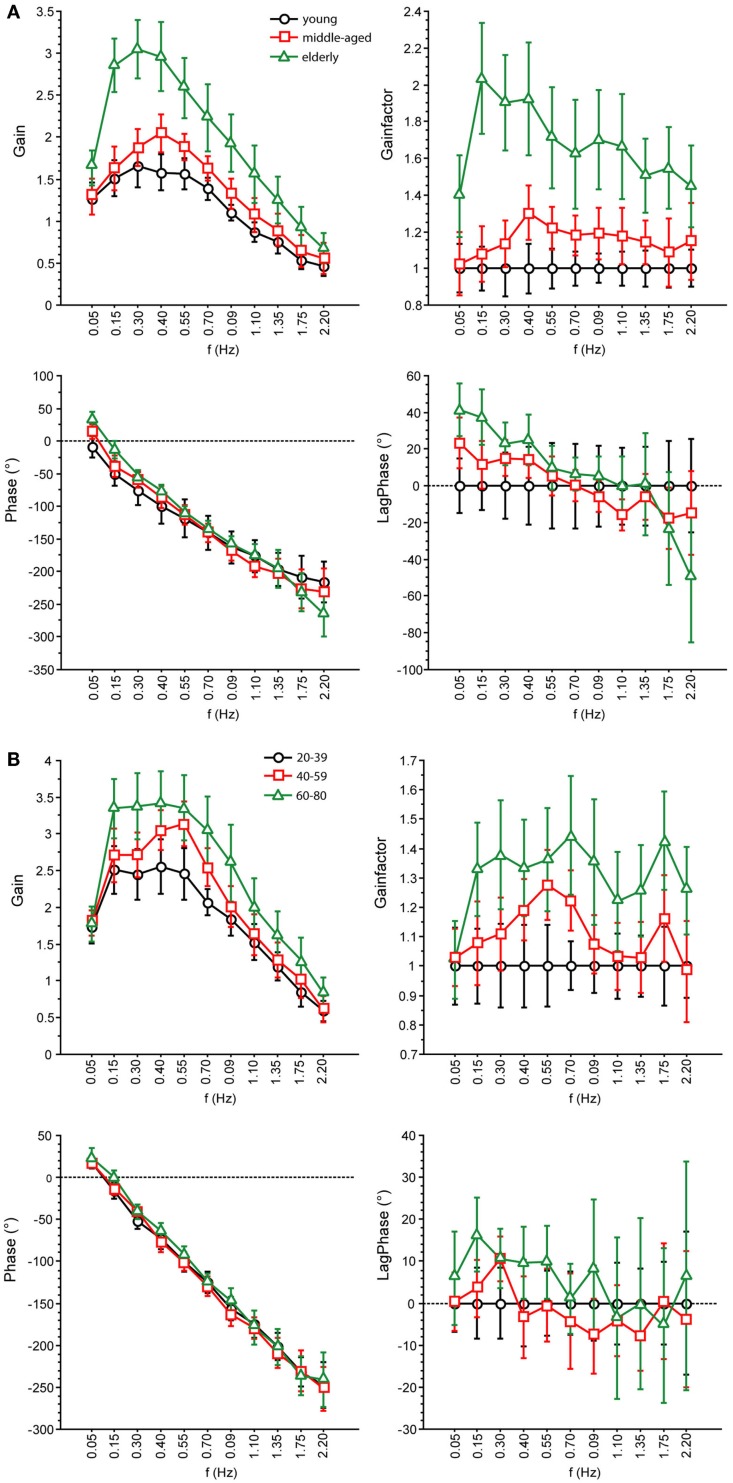
**Parameters of perturbed stance**. GAIN, GAINFACTOR, PHASE, LAGPHASE of the three age groups at 1° with eyes open **(A)** and eyes closed **(B)**.

**Figure 7:**
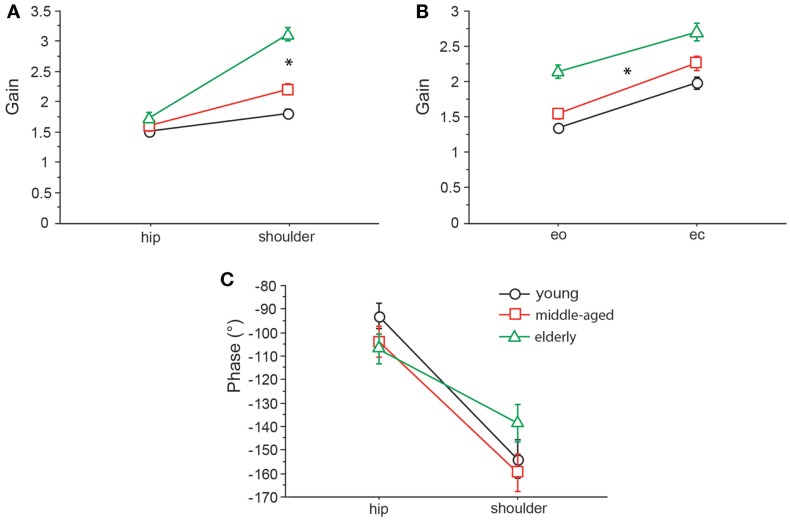
**GAIN interaction between age and body segments and age and visual condition, and PHASE interaction between age and body segments**. GAIN of the three age groups with respect to body segment **(A)** and visual condition **(B)** and PHASE of the three age groups with respect to body segment **(C)**. eo, eyes open; ec, eyes closed.

We found significant differences in GAIN between the three age groups (*F* = 327.5, *p* < 0.0001). In the young group, GAIN was lowest (1.66 ± 0.022) whereas it was highest in the elderly group (2.42 ± 0.021). Across all groups, GAIN was on average 37.6% higher with eyes closed than with eyes open (*F* = 621.2, *p* < 0.0001). GAIN significantly depended on stimulus amplitudes (0.5°: 2.21 ± 0.018, 1°: 1.80 ± 0.018, *F* = 249.3, *p* < 0.0001), on stimulus frequencies (*F* = 394.9, *p* < 0.0001), and on body segments (hip: 1.62 ± 0.018, shoulder: 2.40 ± 0.018, *F* = 878.3, *p* < 0.0001). Age group significantly interacted with frequency (*F* = 9.52, *p* < 0.0001). The major GAIN difference between age groups appeared to be in the lower frequency range, except for the lowest frequency value (see GAIN and GAINFACTOR plots in Figures 5 and 6). Moreover, age group significantly interacted with body segments (*F* = 170.7, *p* < 0.0001). This represents the fact that GAIN of the shoulder in the elderly group was almost twice as large as that of the hip, whereas in the young group, shoulder GAIN was 20% larger than hip GAIN (Figure 7A). Finally, age group significantly interacted with visual conditions (*F* = 3.17, *p* = 0.042), representing the finding that GAIN with eyes closed was about 50% larger than with eyes open in the young group, whereas the GAIN increase with closing the eyes was only 25% in the elderly group (Figure 7B). There was no significant interaction between age group and stimulus amplitude (*F* = 0.9, *p* = 0.39).

Across all age groups, PHASE was mainly determined by frequency (*F* = 849.2, *p* < 0.0001). PHASE differed significantly between the three age groups (young: −123.45 ± 1.52°, middle-aged: −131.82 ± 1.65°, elderly: −122.77 ± 1.48°; *F* = 9.9, *p* = < 0.0001). More specifically, the PHASE profile as a function of frequency was different between the age groups. While the young group showed a moderate slope of the PHASE as a function of stimulus frequencies, the middle-aged and the elderly group presented a steeper relationship between PHASE and frequencies (see Figures 5 and 6). Interestingly, the PHASE profile difference between the middle-aged and the elderly group mainly consisted of a downward shift (reduction of phase lag) that was consistent across all frequencies (LAGPHASE plots in Figures 5 and 6). In general, the effect of age on PHASE as a function of frequency was characterized by a significant interaction between age and frequency (*F* = 3.5, *p* < 0.0001). Across all age groups, phase lag was found to be significantly smaller with eyes closed (−121.82 ± 1.27 °) than with eyes open (−129.44 ± 1.27°, *F* = 17.6, *p* < 0.0001), significantly smaller at the hip (−101.26 ± 1.27°) than at the shoulder level (−150.01 ± 1.27°, *F* = 761.5, *p* < 0.0001), but was not significantly different across different stimulus amplitudes (*F* = 0.02, *p* = 0.87). Age group significantly interacted with body segment (*F* = 27.1, *p* < 0.0001) representing the fact that phase difference between shoulder and hip decreases with age (see Figure 7C). There was no significant interaction between age group and visual condition (*F* = 0.5, *p* = 0.6002).

### Model parameters

**Figure 8:**
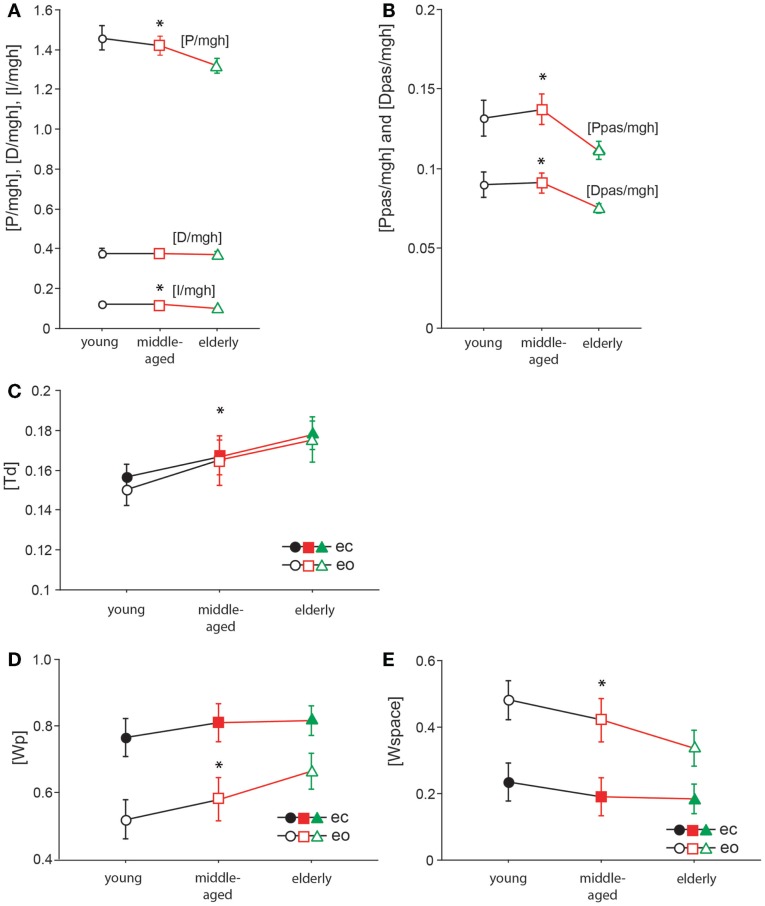
**Model parameters**. [P/mgh] in rad^−1^ **(A)**, [D/mgh] in s × rad^−1^ **(A)**, [I/mgh] in s^−1^ × rad^−1^ **(A)**, [Ppas/mgh] **(B)**, [Dpas/mgh] **(B)**, [Td] in s **(C)**, [Wp] **(D)**, and [Wspace] **(E)** of the three age groups. The three different age groups are shown on the *x*-axis; eo, eyes open; ec, eyes closed.

The model parameter/stiffness factor [P/mgh] is the proportional gain of the neural controller. We found significant differences of [P/mgh] across the three age groups (*F* = 9.3, *p* = 0.0001, see Figure 8A). This parameter was highest in the young group (1.43 rad^−1^) and lowest in the elderly group (1.29 rad^−1^). Across all age groups, [P/mgh] was significantly higher at a stimulus amplitude of 1°(1.41 rad^−1^) than of 0.5°(1.33 rad^−1^, *F* = 6.7, *p* = 0.01), representing a slight amplitude non-linearity of the system. Visual conditions did not significantly influence [P/mgh] (*F* = 0.7, *p* = 0.42). The age group did not significantly interact with visual condition (*F* = 0.9, *p* = 0.39) or stimulus amplitude (*F* = 0.02, *p* = 0.98).

The derivative gain or damping factor, [D/mgh], did not significantly vary with age (*F* = 0.1, *p* = 0.87, Figure 8A). This parameter was nearly equal in the three age groups (young: 0.372 s × rad^−1^, middle-aged: 0.370 s × rad^−1^, elderly: 0.365 s × rad^−1^). ([D/mgh] was significantly higher with eyes closed (0.37 s × rad^−1^) than with eyes open (0.35 s × rad^−1^, *F* = 9.34, *p* = 0.0025). Stimulus amplitude had a significant effect on [D/mgh] (0.5°: 0.36 s × rad^−1^, 1°: 0.38 s × rad^−1^, *F* = 4.4, *p* = 0.04). The age group did not have a significant interaction with visual condition (*F* = 0.47, *p* = 0.62) or stimulus amplitude (*F* = 0.23, *p* = 0.79) considering their effect on [D/mgh]).

The integral gain, [I/mgh], was significantly higher in the young and the middle-aged group (young: 0.118 s^−1^ × rad^−1^, middle-aged: 0.120 s^−1^ × rad^−1^) than in the elderly group (0.097 s^−1^ × rad^−1^, *F* = 9.4, *p* = 0.0001, Figure 8A). Visual condition had a significant effect on [I/mgh] (eo: 0.118 s^−1^ × rad^−1^, ec: 0.104 s^−1^ × rad^−1^, *F* = 8.8, *p* = 0.0033), while stimulus amplitude did not (*F* = 0.02, *p* = 0.88). The age was found to interact significantly with visual condition (*F* = 1.1, *p* = 0.34) or stimulus amplitude (*F* = 0.2, *p* = 0.86) in their effect on [I/mgh].

The age group was found to have a significant effect on the passive stiffness factor [Ppas/mgh] (*F* = 9.9, *p* < 0.0001, see Figure 8B). [Ppas/mgh] was smallest in the elderly group (0.109) and larger in the other two age groups (young: 0.130, middle-aged: 0.135). [Ppas/mgh] was significantly higher with eyes open (0.135) than with eyes closed (0.113, *F* = 19.02, *p* < 0.0001). Stimulus amplitude did not significantly influence [Ppas/mgh] (*F* = 0.6, *p* = 0.45). The age group was not found to significantly interact with visual condition (*F* = 0.5, *p* = 0.62) or stimulus amplitude (*F* = 0.3, *p* = 0.72) with respect to [Ppas/mgh].

[Dpas/mgh], the passive damping factor, decreased significantly with age (*F* = 9.07, *p* = 0.0002, Figure 8B). It was highest in the young group (0.089) and lowest in the elderly group (0.074). [Dpas/mgh] was significantly higher with eyes open (0.089) than with eyes closed (0.078, *F* = 10.9, *p* = 0.0011). Stimulus amplitude had no significant influence on [Dpas/mgh] (*F* = 0.3, *p* = 0.61). The age group was not observed to have a significant interaction with visual condition (*F* = 0.14, *p* = 0.87) or stimulus amplitude (*F* = 0.1, *p* = 0.89) with respect to [Dpas/mgh].

Time delay, [Td], significantly increased with age (*F* = 14.1, *p* < 0.0001, Figure 8C). It was lowest in the young group (0.153 ± 0.003 s) and highest in the elderly group (0.177 ± 0.003 s). Neither visual condition (*F* = 0.9, *p* = 0.34) nor stimulus amplitude (*F* = 2.9, *p* = 0.09) had a significant effect on [Td]. Moreover, age group did not significantly interact with visual condition (*F* = 0.2, *p* = 0.85) or stimulus amplitude (*F* = 0.88, *p* = 0.42) with respect to [Td].

The proprioceptive sensory weight, [Wp], increased significantly with increasing age (*F* = 7.2, *p* = 0.0009, see Figure 8D). Being lowest in the young group (0.64 ± 0.018) and highest in the elderly group (0.74 ± 0.018), [Wp] was 35% higher with eyes closed (0.8 ± 0.015) than with eyes open (0.59 ± 0.015), and this difference was significant (*F* = 93.5, *p* < 0.0001). The stimulus amplitude significantly affected [Wp] (0.5°: 0.74 ± 0.015, 1°: 0.65 ± 0.015, *F* = 18.7, *p* < 0.0001), resulting in a 14% increase. The age group did not significantly interact with visual condition (*F* = 1.8, *p* = 0.16) or stimulus amplitude (*F* = 0.3, *p* = 0.77) in their effect on [Wp]. In addition to [Wp], we show a figure of [Wspace] (Figure 8E). [Wspace] is 1-[Wp] and it reflects vestibular weight with eyes closed and visual and vestibular weight with eyes open.

### Clinical tests

The elderly group performed also two clinical tests: the TUG and the FRT. The average results were for the FRT a reach distance of 28.2 ± 6.4 cm and for the TUG 8.48 ± 1.2 s.

**Figure 9:**
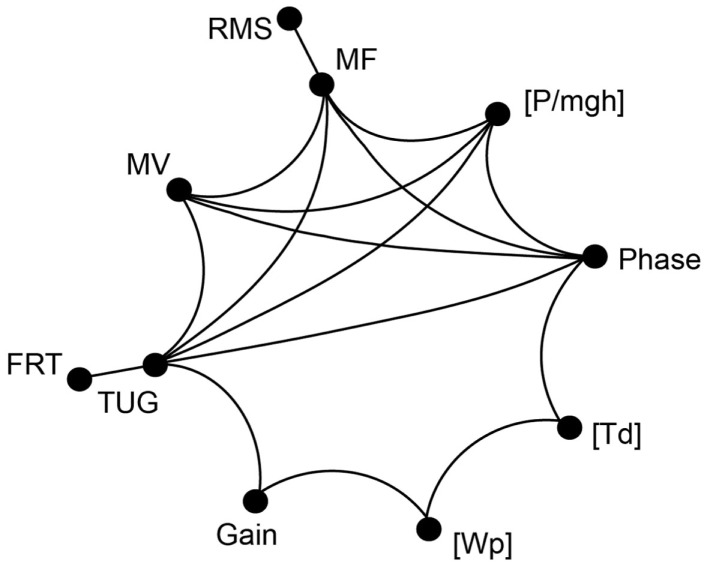
**Correlation of clinical tests and measurements of spontaneous and perturbed stance**. Correlation matrix between clinical test parameters and parameters obtained from platform measures. The correlation revealed a group of parameters that all significantly correlate with each other (MF, MV, PHASE, [P/mgh], and TUG). Some of the measures mentioned above significantly correlate with additional measures and parameters. Only correlations with *R* > 0.47 and *p* < 0.05 are shown.

In the following, we report significant correlations between our experimental spontaneous sway measures and measures of perturbed stance, derived model parameters, and the results of clinical tests in the elderly group (see Figure 9; Table 1). However, we calculated the correlation matrix only between measures (spontaneous and perturbed sway measures) and parameters that were significantly different from young subjects. PHASE, MF, MV, [P/mgh], and TUG all correlated significantly with each other. In addition, PHASE correlated significantly with [Td], [Td] correlated with [Wp], [Wp] correlated with GAIN and GAIN correlated with TUG. By contrast, RMS correlated only with MF and FRT correlated only with TUG.

**Table 1:** **Significance of correlations in terms of *p*-values (*p* < 0.05) and the associated *R*-values between experimental spontaneous sway measures and measures of perturbedstance, derived model parameters, and the results of the clinical tests in the elderly group**.

	Phase	[P/mgh]	MF	RMS	MV	TUG	FRT	GAIN	[Wp]	[Td]
**Phase**										
[P/mgh]	*p* = 0.0008									
	*R* = 0.72	
MF	*p* = 0.0001	*p* = 0.0001								
	*R* = 0.79	*R* = 0.88	
RMS	n.s.	n.s.	*p* = 0.0307							
			*R* = −0.51	
MV	*p* = 0.0012	*p* = 0.0028	*p* = 0.0052	n.s.						
	*R* = 0.70	*R* = 0.66	*R* = 0.63	
TUG	*p* = 0.0369	*p* = 0.002	*p* = 0.0353	n.s.	*p* = 0.0464					
	*R* = 0.49	*R* = 0.68	*R* = 0.50		*R* = 0.47	
FRT	n.s.	n.s.	n.s.	n.s.	n.s.	*p* = 0.0433				
						*R* = −0.48	
GAIN	n.s.	n.s.	n.s.	n.s.	n.s.	*p* = 0.0461	n.s.			
						*R* = 0.48	
[Wp]	n.s.	n.s.	n.s.	n.s.	n.s.	n.s.	n.s.	*p* = 0.0001		
								*R* = 0.78	
[Td]	*p* = 0.0343	n.s.	n.s.	n.s.	n.s.	n.s.	n.s.	n.s.	*p* = 0.0331	
	*R* = −0.51								*R* = 0.50	

*n.s., not significant; MF, mean frequency; RMS, root mean square; TUG, timed up and go test; FRT, functional reach test; Td, time delay*.

## Discussion

We analyzed postural control of elderly people (60–80 years) and compared their data to the data of two younger groups (20–39 and 40–59 years). Postural control was characterized by spontaneous sway measures and measures of perturbed stance between 0.05 and 2.2 Hz. Perturbations were induced by a pseudorandom platform tilt stimulus. Stimulus–response data were interpreted on the basis of a simple negative feedback model (Peterka, 2002; Maurer et al., 2006a; Engelhart et al., 2014).

Among the spontaneous sway measures, RMS, MV, and MF were significantly higher in elderly people (elderly group) than in young people (young group). These results are in line with previous studies (Maki et al., 1990; Hytönen et al., 1993; Baloh et al., 1994; Collins et al., 1995; Prieto et al., 1996; Tang and Woollacott, 1996; Maurer and Peterka, 2005). The effect on MV had the highest significance. The high sensitivity of the MV effect as compared to other spontaneous sway measures to detect abnormalities was reported earlier (Prieto et al., 1996; Maurer and Peterka, 2005; Ruhe et al., 2010; Delignières et al., 2011; Moghadam et al., 2011). In previous work by our group (Maurer and Peterka, 2005) and others (Maki et al., 1990; Prieto et al., 1996), higher MV of elderly people was interpreted as a higher amount of regulatory balancing activity. Furthermore, MV was different from other spontaneous sway parameters as the age effect significantly interacted with sway direction (a–p vs. m-l). The interaction with sway direction was based on the fact that elderly’s MV in the a–p direction was much larger (+36%) than in m-l direction, whereas this directional effect was much weaker in the younger groups. As already shown in other studies, a–p sway might be more sensitive to any impairment (Collins et al., 1995; Prieto et al., 1996; Tia et al., 2012), due to the fact that balance in a–p direction follows the rules of an unstable inverted pendulum.

Mean velocity of the middle-aged and elderly group increased about 30% (elderly: 26%, middle-aged 36%) across all body segments when closing the eyes. In the young group, this increase amounted to only 18%. Independently of age, the increase of MV when closing the eyes has been described before [e.g., Era and Heikkinen (1985), Horak et al. (1989), Teasdale et al. (1991), Hytönen et al. (1993), Prieto et al. (1996), Accornero et al. (1997), Schieppati et al. (1999), and Shumway-Cook and Woollacott (2001)]. It is still controversial whether this increase is more or less pronounced in elderly than in young subjects. Peterka and Black (1990) and Teasdale et al. (1991) observed as well no significant age-related increase in spontaneous sway with eyes closed. Hytönen et al. (1993) found an increased difference of sway velocity between eyes closed and eyes open in their eldest group (76–90 years).

In general, spontaneous sway measures in our study seemed to mirror age-dependent changes of postural control in a reliable way. However, the variance across scores of different spontaneous sway measures is known to be covered by only two principal components, even if one analyzes up to 14 different measures [see Maurer and Peterka (2005)]. That might be the reason why measuring spontaneous sway does not allow for detecting specific constituents of postural control deficits (Horak et al., 1989; Maki et al., 1990; Tang and Woollacott, 1996; Kuo et al., 1998; Black, 2001; Ghulyan et al., 2005). From a model-based perspective, many different sources of postural control deficits like, e.g., increased feedback time delay, too strong or too weak feedback gain, increased sensory noise level, and abnormal weighting of sensory inputs may all lead to increased sway or sway velocity (Maurer and Peterka, 2005). For a more specific analysis of postural control deficits, an external perturbation is required (Engelhart et al., 2014). Consequently, we characterized subjects’ behavior as a function of external perturbations, i.e., anterior–posterior platform tilts, by transfer functions in the frequency domain. Transfer functions consist of gain and phase curves. We first discuss GAIN and PHASE findings separately before we integrate the findings using our model-based approach.

GAIN was highest in elderly and lowest in young people, which is in line with earlier findings [e.g., Ghulyan et al. (2005) during sinusoidal platform translations]. Across all age groups, GAIN significantly depended on visual conditions, stimulus amplitudes, stimulus frequencies, and on body segments. Moreover, we found significant interactions between age, on one hand, and frequency, visual condition, and body segments, on the other. The interaction between age and frequency represents the fact that the major GAIN difference between age groups appeared in the lower frequency range. The interaction between age and visual condition is due to the fact that the GAIN increase was about 48% when closing the eyes in the youngest group and only 26% in the elderly group. The significant interaction between age and body segments is related to the relatively larger shoulder GAIN (almost twice as large as the hip GAIN) in elderly people, whereas in young people, shoulder GAIN was 20% larger than hip GAIN. This finding is in line with the fact that elderly people are known to have a tendency to engage hip flexion/extension when stance is perturbed (Kuo et al., 1998; Shumway-Cook and Woollacott, 2001).

PHASE as a function of frequency was significantly different between the three age groups. The PHASE decreased with increasing frequency in all age groups; however, the middle-aged and the elderly group displayed a steeper relationship between PHASE and frequencies. Age significantly interacted with body segment, representing the fact that PHASE differences between shoulder and hip decreases with age.

In order to interpret the numerous findings concerning GAIN and PHASE curves, we fitted subjects’ data by a simple feedback system that is known to adequately describe body motion as a function of sensory inputs [e.g., Mergner et al. (2002, 2003), Vette et al. (2010), Van der Kooij and Peterka (2011), and Engelhart et al. (2014)]. A very basic version consists of the body represented by mass and height, a neural controller including stiffness factor and damping factor, a feedback time delay, and sensory weighting mechanism that integrates proprioceptive, vestibular, and visual cues (Maurer et al., 2006a; Van der Kooij and Peterka, 2011). We had to reject our hypothesis that elderly people show a general decline in sensory, motor, and higher-level adaptation systems. The most significant differences between age groups relate to the sensory weighting, the feedback gain of the neural controller, and the overall time delay of the motor reaction: Across all stimulus conditions, elderly people weigh proprioceptive cues ([Wp]) higher than visual and vestibular cues. This finding suggests that elderly people tend to stabilize and orient their body relative to the support surface and, therefore, rely more on proprioceptive than on vestibular or visual cues, which is related to the larger GAIN across all frequencies. Abnormalities in the use of sensory cues have already been reported by others (Nishihori et al., 2012; Maitre et al., 2013). All subjects weigh proprioceptive cues relatively stronger when they close their eyes, i.e., one space reference cue (visual) is missing. This relates to the larger GAIN observed with eyes closed than with eyes open. The vestibular cue as another space cue does not fully compensate for the lack of the visual cue as also reported by others (Ishida et al., 1997). This is in line with the experience of other laboratories that other cues fill in when sensory cues are missing (Van der Kooij and Peterka, 2011).

Although not being significant, elderly people tend to down-regulate proprioceptive cues when opening the eyes less than young people do. Then again, all age groups similarly down-regulate their proprioceptive weights with increasing stimulus amplitude, representing the fact that with larger disturbances of the support surface, stabilization in space becomes advantageous to avoid large body sway (Peterka, 2002; Van der Kooij and Peterka, 2011). This might indicate that the reliability of the sensory weighting process is not impaired in elderly people as a result of aging.

Elderly people display an overall time delay that is about 24 ms longer than that of young people. Our results are almost identical to the results of Davidson et al. (2011) in which the time delay of the motor reaction in elderly people was found to be 23 ms higher than in the young people when perturbing stance with jolts of a ballistic pendulum. A large time delay endangers stability of the system through an enlarged oscillation tendency (Peterka, 2000; Van der Kooij and Peterka, 2011). Since a high-proportional feedback gain increases oscillation tendencies, too, a lower feedback gain in elderly might represent a compensation strategy to reduce oscillations (Peterka, 2002; Van der Kooij and Peterka, 2011). In fact, we identified smaller feedback gain parameters (active proportional, [P/mgh]; passive proportional, [Ppas/mgh]; passive damping factor, [Dpas/mgh]) in elderly compared to young subjects.

Everyday postural control is usually assessed by simple clinical tests and scales such as the TUG (Podsiadlo and Richardson, 1991; Enkelaar et al., 2013) and the FRT (Duncan et al., 1990; Enkelaar et al., 2013). In order to relate our findings to established measures of postural control, our elderly subjects performed FRT and TUG. The FRT revealed an average reach distance of 28.2 cm in the elderly group. The reach distance of our elderly group is similar to data of Duncan et al. (1990) who evaluated the FRT in 128 volunteers between 21 and 87 years. Their group of elderly people aged between 70 and 87 years attained a reach distance of 25.0 cm whereas the younger group (20–40ys) showed a distance of 39.8 ± 5.2 cm. In our study, the TUG in the elderly group amounted to 8.48 s. The TUG scores of the elderly group are similar to the one reported by Buatois et al. (2006); (9.6–10.2 s), Nagy et al. (2007); (8.9–10.3 s), Enkelaar et al. (2013); (9.3 s), and Bohannon (2006); (9.2 s).

The correlation analysis between clinical scales (FRT, TUG) and our experimental findings in the elderly group revealed a group of parameters that all significantly correlated with each other, consisting of two spontaneous sway measures (MF, MV), one measure of perturbed stance (PHASE), one model-derived parameter ([P/mgh]), and the clinical measure of TUG (see Table 1). This could be explained by the fact that all those parameters may be influenced by systems stability. Why would a clinical score like TUG, which involves walking and transitioning between straight walking, turning, sit-to-stand and stand-to-sit motions, correlate with parameters that describe human postural control? We assume that stability of the postural control system also affects more complex postural tasks such as walking or certain transitions.

Furthermore, there was a significant correlation between PHASE and [Td], which are both related to the timing of a response following a stimulus. [Wp] correlated significantly with GAIN and this is consistent with [Wp] being responsible for a uniform scaling of the gain curves across all frequencies.

It was interesting to find that the FRT correlated only with the TUG. The fact that the FRT seems to correlate with other clinical balance measures has been reported by others (Granacher et al., 2009; Enkelaar et al., 2013: moderate correlation). However, it is not clear why a stance parameter related to the amount of forward voluntary lean is related to a complex dynamic movement task as the TUG. We deem it likely, however, that the endangered stability of the postural control system described above, might influence both, voluntary lean and more complex movement tasks. Further investigations are needed to explain this finding. Furthermore, RMS correlated significantly solely with MF, while MF correlated with many other measures of perturbed sway analogously to MV, supporting our view that spontaneous sway measures are gross measures related to balance control and that the information contained in those measures is highly correlated and possibly redundant (Maurer and Peterka, 2005). More specifically, sway amplitude, represented by RMS, might be less crucial for the postural stability than velocity related measures such as MV and MF.

In summary, the perturbation-based approach presented here gives us more insight about postural control mechanisms of elderly people than measures of spontaneous sway do. In addition, it allows for a model-based interpretation of the experimental data, which provide valuable information of underlying mechanisms of the whole postural control system. We were able to identify basic parameters of the postural control system that are related to aging, i.e., the increased reliance of elderly people on proprioception, the decreased feedback loop gain, and the increased closed loop feedback time delay between the sensory perception and the motor reaction. Understanding the postural changes during aging is crucial for the development of new therapies that improve postural control in the elderly population. Our approach is highly sensitive even to small changes in postural control. Accordingly, it might be also suited to monitor therapeutic interventions, which aim to improve postural stability in the elderly.

## Conflict of Interest Statement

The authors declare that the research was conducted in the absence of any commercial or financial relationships that could be construed as a potential conflict of interest.
